# Application of modified endotracheal tube in nursing of retracted stoma with severe moisture-associated skin damage: A case report

**DOI:** 10.1097/MD.0000000000046195

**Published:** 2025-11-28

**Authors:** Hang Ruan, Liang Lv, Zhan Shen, Jinmin Shen

**Affiliations:** a Department of Gastrointestinal and Colorectal Surgery, Shulan (Hangzhou) Hospital Affiliated to Zhejiang Shuren University Shulan International Medical College, Hangzhou, Zhejiang, China; b Department of Anorectal Surgery, Shulan (Anji) Hospital, Huzhou, Zhejiang, China.

**Keywords:** endotracheal tube modification, fecal diversion, moisture-associated skin damage, stoma retraction

## Abstract

**Rationale:**

Severe stomal retraction often leads to peristomal moisture-associated skin damage (MASD), posing a significant challenge to conventional ostomy care, particularly for critically ill patients who are not candidates for surgical revision. To address this, we used an innovatively modified tracheal tube for fecal diversion. This approach not only effectively diverted fecal output but also prevented further skin exposure, thereby creating an environment conducive to healing MASD.

**Patient concerns:**

A 40-year-old male with metastatic colon cancer and a retracted colostomy developed a 3 cm × 3 cm refractory ulcer with persistent leakage despite standard care, causing significant pain and impaired quality of life.

**Diagnoses:**

Severe peristomal ulceration with stoma retraction, metastatic colon adenocarcinoma, and critical complications including respiratory failure and pneumonia.

**Interventions:**

A modified endotracheal tube was used for fecal diversion. The tube was customized by truncating the distal end and sealing the lumen, then inserted and secured via cuff inflation. Complementary care included topical wound management and dietary control to maintain semiliquid stool.

**Outcomes:**

The peristomal ulcer achieved complete epithelialization within 10 days. The patient reported substantial alleviation of discomfort and high satisfaction with the treatment, and nursing efficiency improved without complications.

**Lessons:**

This technique offers a simple, cost-effective, and nonsurgical option for fecal diversion in complex stoma-related MASD, especially valuable in critical or resource-limited settings, though ongoing monitoring is essential to prevent tube obstruction.

## 1. Introduction

In North America, approximately 70% of patients who undergo ostomy experience complications of the stoma or peristomal area, among which peristomal skin damage is the most common type.^[1]^ Peristomal moisture-associated skin damage (MASD) is the most prevalent type of peristomal skin damage.^[2,3]^ Patients with a retracted or collapsed stoma often experience poor adherence of the ostomy bag baseplate to peristomal skin. Owing to the resulting gap, stool can easily leak from the stoma base, potentially leading to MASD, including infection, ulceration, and even bleeding. When this occurs, it can cause significant patient discomfort, reduce the ostomy bag lifespan, and increase the time and effort required for stoma care. For most mild to moderate stoma retraction issues, the clinical routine is to use convex ostomy bags in combination with barrier paste, which effectively prevents fecal leakage. In cases of severe retraction, stoma care is significantly more challenging. Such cases often require surgical stoma reconstruction. In clinical practice, we encounter patients who are unsuitable for stoma revision surgery because of various contraindications, particularly those with critical illness in the intensive care unit or significant frailty. Even with professional stoma care, MASD may develop. Once MASD occurs, healing is often prolonged and challenging. For patients with MASD who are not surgical candidates, the treatment options remain limited.

In this study, we introduced an innovative approach using a modified tracheal cannula to divert fecal flow in a patient with advanced colon cancer complicated by severe peristomal MASD due to stoma retraction. This method effectively prevents prolonged exposure of the peristomal skin to fecal leakage, creating an optimal environment for wound healing. In addition, it significantly alleviated patient discomfort and reduced the burden of clinical nursing care. Our findings offer a valuable reference for the management of similar cases in clinical practice.

## 2. Case presentation

### 2.1. General information

A 40-year-old Chinese male with advanced colon cancer initially underwent laparoscopic radical right hemicolectomy on December 27, 2018, for hepatic flexure colon cancer (postoperative pathology: pT4N1M0). Genetic testing revealed a BRAF V600E mutation in the wild-type KRAS. Adjuvant chemotherapy (the XELOX regimen) was administered postoperatively.

Two years later, surveillance imaging revealed pelvic metastasis. Despite multimodal therapy including radiotherapy, chemotherapy (FOLFOX/FOLFIRI), targeted therapy (cetuximab/bevacizumab), and immunotherapy (pembrolizumab), disease progression persisted, with tumor invasion into the rectum and seminal vesicles. Consequently, total mesorectal excision and bilateral seminal vesiculectomy were performed on December 21, 2020.

**Figure 1.**
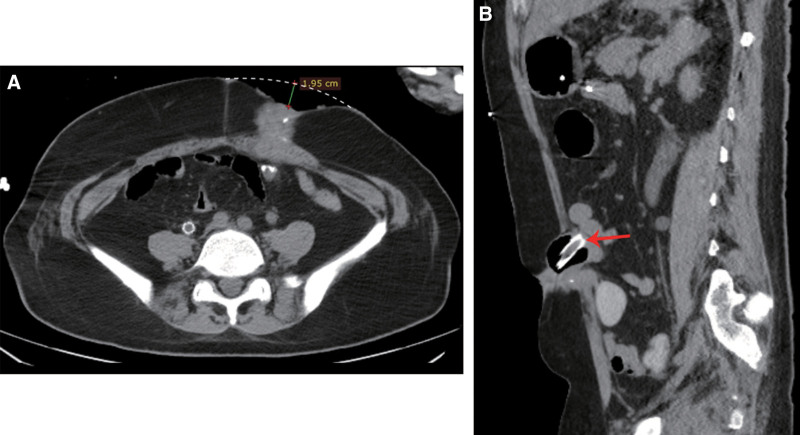
(A) Abdominal computed tomography (CT) revealed significant retraction of the sigmoid colostomy. (B) Follow-up CT revealed the modified endotracheal tube maintained excellent sealing and full intestinal wall contact. CT = computed tomography.

**Figure 2.**
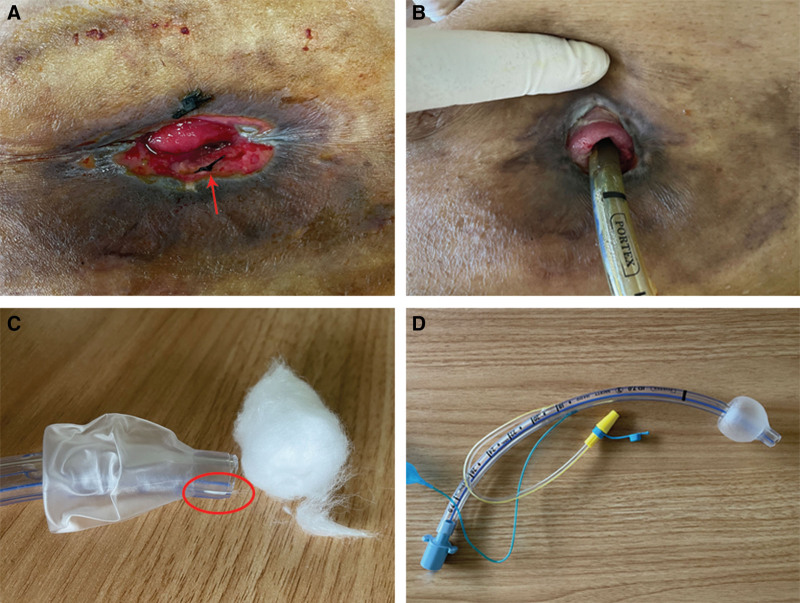
(A) Examination revealed a 3 cm × 3 cm ulcerated lesion on the peristomal skin. (B) The peristomal skin ulcer has healed following treatment. (C) A composite of cotton wool and cyanoacrylate adhesive was used to achieve complete sealing of the endotracheal tube’s inner lumen. (D) Inflate the endotracheal tube cuff and check for air leaks.

Following the procedure, the patient developed a rectal anastomotic fistula refractory to conservative management and subsequent surgical repair attempts. Concurrently, the pelvic tumor demonstrated progressive growth complicated by recurrent urinary tract infections and secondary perianal abscess formation. Definitive management was achieved on January 21, 2021, through sigmoid colostomy with perianal abscess incision and drainage, which resulted in clinical improvement. Despite the ongoing standard chemotherapy and targeted therapy regimens, the malignancy exhibited continued progression with metastatic spread. The patient subsequently developed severe pneumonia manifesting as hemoptysis and significant hypoxemia (SpO_2_ < 90%), which required urgent transfer to the intensive care unit on April 8, 2024. Two weeks later, the patient developed persistent bleeding from a peristomal skin ulcer that was managed with bedside suture hemostasis. Final diagnoses: metastatic colon adenocarcinoma (pulmonary, abdominal, and pelvic metastases), pulmonary hemorrhage, pulmonary infection, respiratory failure, kidney injury. heart failure. and hypertension. Physical examination: The patient was conscious and hemodynamically stable. The sigmoid colostomy in the left lower quadrant showed marked retraction (approximately 2 cm in depth, Fig. 1A) while maintaining patency, as evidenced by active passage of flatus and stool. A 3 cm × 3 cm ulcerated lesion was observed in the peristomal skin (Fig. 2A). The stoma demonstrated mild stenosis but remained traversable by an index finger. After the utilization of the convex ostomy bag and the application of the barrier paste, fecal leakage from the patient’s stoma persisted. As a result, the ostomy bag had to be replaced at frequent intervals. Moreover, the skin ulceration around the stoma inflicted severe pain and discomfort on the patient.

The study has been approved by the Research Ethics Committee of Shulan (Hangzhou) Hospital. The patient has provided written informed consent for this study and report.

### 2.2. Tracheal tube modification

First, a digital examination of the stoma was performed to verify patency. Next, an appropriate model of the endotracheal tube was selected based on the stoma’s diameter. Subsequently, the head of the endotracheal tube – including the Murphy eye and bevel – was cut as close as possible to the cuff. To prevent air leakage from the transected tube, the inner lumen was sealed using a combination of cyanoacrylate adhesive and cotton wool; the cotton matrix expedited the adhesive’s curing (Fig. 2C). Following this, the absence of air leakage was confirmed (Fig. 2D).

The procedure continued by fully deflating the cuff, after which the endotracheal tube was inserted through the stoma. Once the cuff was inflated, the tube was gently pulled back, ensuring its sealing effect prevented fecal leakage around the tube (Fig. 1B). Finally, a plastic bag could be attached to collect stool, further simplifying nursing care.

### 2.3. Post-procedure care

For ulcerated skin around the stoma, the dressings should be changed regularly. Additionally, topical anti-inflammatory sprays, such as triamcinolone, may be used to decrease peristomal skin inflammation and enhance healing.^[4]^ A layer of sterile petrolatum gauze should be applied to protect the stoma periphery from stool exposure, followed by an absorbent gauze dressing to manage potential effluent seepage, facilitating moist wound healing of the skin surrounding the stoma. Change dressings immediately if fecal leakage is observed.

### 2.4. Outcomes

Following 10 days of treatment, the peristomal ulcer showed complete healing (Fig. 2B). The associated pain and discomfort had resolved, and the patient reported no significant adverse events throughout the recovery period, expressing a high level of satisfaction with the treatment.

## 3. Discussion

Peristomal MASD typically results from prolonged exposure of the enterostomy-surrounding skin to fecal effluent beneath the ostomy device. This condition frequently presents as concurrent skin breakdown, infection, and ulceration. Given that small-intestinal effluent contains higher moisture content and abundant active digestive enzymes, patients with small-bowel stomas demonstrate a significantly higher incidence of peristomal MASD than those with colostomies.^[5,6]^ Research indicates that peristomal MASD risk factors include age, diabetes, stoma retraction, peristomal folds, abdominal radiotherapy history, chemotherapy exposure, liquid feces, and improper stoma care.^[7]^ Stoma retraction is a significant challenge in clinical nursing practice. Subsequent complications, including peristomal skin infections, ulcerations, and even hemorrhagic lesions, typically demonstrate delayed healing. These conditions not only inflict physical discomfort, but also substantially impact patients’ psychological well-being, frequently manifesting as depression and anxiety. Moreover, such complications may necessitate treatment modifications, ultimately compromising overall patient prognosis.^[8]^ A relatively common method is to use leak-proof paste or strips combined with convex baseplates to reduce the gap between the baseplate and the stoma skin to reduce the leakage of fecal fluid. Despite proper application, patient movement and positional changes can disrupt the baseplate-stoma seal, creating gaps that permit effluent leakage in cases of stoma retraction. Furthermore, stoma retraction significantly increases nursing complexity, necessitating more frequent appliance changes and prolonging each procedure.

In this clinical case, the required materials are readily available, and the production process is straightforward while demonstrating satisfactory therapeutic outcomes. This approach demonstrates particular advantages for inexperienced departments, as tracheal intubation eliminates the need for subsequent ostomy appliance changes, thereby reducing the stoma care requirements. Hua et al^[9]^ conducted a clinical study involving 149 patients who underwent low colorectal or coloanal anastomosis with ileal diversion using endotracheal tubes. Their findings demonstrated that cannula ileostomy is a safe and effective diversion technique for protecting low colorectal and coloanal anastomoses. Clinically, the sharp tip of the endotracheal tube may angulate due to positional changes, leading to poor contact with the intestinal wall. Moreover, there is a potential risk of intestinal wall damage and perforation. Trimming the distal end of the endotracheal tube improves fecal diversion and minimizes the risk of intestinal trauma. Additionally, adequate exposure of the peristomal skin establishes an optimal healing microenvironment while enhancing accessibility of nursing care. However, owing to the limited lumen and lack of elasticity of the endotracheal tube, it is necessary to provide the patient with a low-residue diet to ensure that their stool remains semiliquid. This is because the feces formed can readily cause lumen obstruction. Therefore, for patients with colostomy, maintenance of semiliquid stool consistency should be achieved through lactulose or polyethylene glycol administration, increased fluid intake, or the use of enteral nutrient solutions. In cases of tube occlusion, initial management should include warm water irrigation. If this was unsuccessful, endotracheal tube replacement was indicated. Notably, the appropriate selection of endotracheal tube size based on stoma diameter is crucial to prevent intestinal compression or fecal leakage. Furthermore, excessive cuff inflation may compress the intestinal wall and induce localized ischemia. Therefore, appropriate inflation with adequate gas or liquid volume is essential.^[10]^

The use of endotracheal tubes for fecal diversion in patients with peristomal MASD has streamlined stoma management. In the present case, the patient with a retracted stoma experienced substantial clinical challenges prior to the intervention, including persistent fecal leakage leading to severe cutaneous irritation and significant psychosocial distress. The necessity for frequent ostomy appliance changes (multiple times daily) imposes considerable physical and emotional burden. Following implementation of the modified endotracheal tube system, clinical outcomes improved markedly, and the patient reported an immediate reduction in effluent leakage, followed by progressive peristomal skin reepithelialization within 7 days, accompanied by substantial pain alleviation. This technique is particularly valuable for clinical units with limited stoma care resources, offering a safe, straightforward, and practical solution using readily available materials. This method is applicable for both retracted and normal stomas. The risk of peristomal fecal dermatitis is significantly reduced by the effective sealing provided by the airbag. Daily care involves cleaning the surrounding skin and reapplying the ostomy bag once dermatitis has fully resolved. This approach not only minimizes the stoma care workload but also alleviates patient discomfort. Thus, the patient can receive enteral nutrition and overall treatment without compromising.

## 4. Limitations

This study has several limitations that should be considered. First, the findings are based on a single-case report, which inherently limits their generalizability and necessitates validation in larger, controlled cohort studies. Second, the use of an endotracheal tube with a narrow lumen presents a potential risk of obstruction, highly dependent on meticulous management of stool consistency. Finally, while no short-term complications were observed, the long-term safety and risks of complications, such as pressure necrosis from the inflatable cuff, remain uncertain and warrant investigation through extended follow-up studies.

## 5. Conclusions

For patients with peristomal MASD, particularly those with a recessed stoma, the modified endotracheal tube technique provides a simple, safe, and feasible short-term fecal diversion method. This approach effectively protected peristomal skin and promoted wound healing.

## Acknowledgments

The authors thank the patient and their family for authorizing the report.

## Author contributions

**Conceptualization:** Hang Ruan.

**Formal analysis:** Liang Lv, Zhan Shen.

**Investigation:** Hang Ruan, Liang Lv.

**Methodology:** Hang Ruan, Zhan Shen, Jinmin Shen.

**Project administration:** Hang Ruan, Jinmin Shen.

**Supervision:** Jinmin Shen.

**Writing – original draft:** Hang Ruan, Zhan Shen.

**Writing – review & editing:** Jinmin Shen.
